# Inhibitory Effects of Decursin Derivative against Lipopolysaccharide-Induced Inflammation

**DOI:** 10.3390/ph17101337

**Published:** 2024-10-07

**Authors:** Jinhee Lee, Jong-Beom Heo, Sanghee Cho, Chang-Woo Ryu, Hae-Joon Heo, Mi-Young Yun, Gaewon Nam, Gyu-Yong Song, Jong-Sup Bae

**Affiliations:** 1 Research Institute of Pharmaceutical Sciences, College of Pharmacy, Kyungpook National University, Daegu 41566, Republic of Korea; aadd8563@gmail.com (J.L.); sanghee3472@naver.com (S.C.); ryu76kr@daum.net (C.-W.R.); 2 College of Pharmacy, Chungnam National University, 99 Daehak-ro, Yuseong-gu, Daejon 34134, Republic of Korea; songmeom@gmail.com (J.-B.H.); boymatt@naver.com (H.-J.H.); 3 Department of Beauty Science, Kwangju Women’s University, Gwangju 62396, Republic of Korea; beauty@kwu.ac.kr; 4 Department of Bio-Cosmetic Science, Seowon University 377-3, Musimseoro, Seowon-gu, Cheongju, Chungbuk 28674, Republic of Korea; skarod@gmail.com

**Keywords:** JB-V-60, endothelium, iNOS, p-STAT-1

## Abstract

Background: This study aims to explore the protective role of JB-V-60—a novel synthetic derivative of decur-sin—against lipopolysaccharide (LPS)-induced inflammation. Methods: We examined the effects of JB-V-60 on heme oxygenase (HO)-1, cyclooxygenase (COX)-2, and inducible nitric oxide synthase (iNOS) in LPS-activated human pulmonary artery endothelial cells (HPAECs). Additionally, we assessed its effects on iNOS, tumor necrosis factor (TNF)-α, and interleukin (IL)-1β in LPS-exposed mice. Results: JB-V-60 enhanced HO-1 levels, inhibited NF-κB activation, reduced COX-2/PGE2 and iNOS/NO concentra-tions, and lowered phosphorylation of signal transducer and activator of transcription 1. It also promoted the translocation of Nrf2 into the nucleus, allowing its binding to antioxidant response elements and resulting in reduced IL-1β in LPS-stimulated HPAECs. The reduction in iNOS/NO levels by JB-V-60 was reversed when HO-1 was inhibited via RNAi. In the animal model, JB-V-60 sig-nificantly decreased iNOS expression in lung tissues and TNF-α levels in bronchoalveolar lavage fluid. Conclusions: These findings highlight the anti-inflammatory effects of JB-V-60 and its potential as a treat-ment for inflammatory disorders.

## 1. Introduction

Heme oxygenase-1 (HO-1) plays a crucial role in protecting against damage from inflammation and the accumulation of reactive oxygen species, particularly in severe conditions such as acute autoimmune disorders, lung diseases, and cancers [1,2]. HO-1 reduces the production of key pro-inflammatory cytokines and mediators, including tumor necrosis factor (TNF)-α, interleukin (IL)-1β, and IL-6 [2]. Studies show that HO-1 offers significant protection to mice against acute septic inflammation and various inflammation-related vascular conditions [2,3]. The Keap1-Nrf2-antioxidant response element (ARE) pathway is crucial for managing oxidative stress and mitigating inflammatory responses by regulating antioxidant and detoxification genes that break down carcinogens and toxins [4,5]. Nrf2 plays a central role in coordinating protective cellular responses to external stressors, making the Nrf2-ARE pathway a key target for treating inflammation-related diseases [4,5]. Furthermore, the signal transducer and activator of transcription (STAT)-1 and nuclear factor (NF)-κB pathways are central to inflammatory signaling [6,7]. When activated, STAT1 and NF-κB stimulate the production of pro-inflammatory molecules that recruit immune cells to inflammation sites [6,7]. However, when dysregulated, these pathways can lead to chronic inflammation, tissue damage, and diverse diseases, underscoring the therapeutic potential of targeting STAT-1 and NF-κB signaling in inflammation-related conditions.

Respiratory dysfunction in the lungs is a severe condition characterized by inflammation, causing swelling, low oxygen levels, and the formation of neutrophil extracellular traps within lung tissues [8]. Lipopolysaccharide (LPS) drives lung diseases by triggering the production of pro-inflammatory mediators through the activation of transcription factors. These molecules sustain chronic inflammation and contribute to the development of inflammatory diseases such as vascular dysfunction, asthma, chronic obstructive pulmonary disease, and cystic fibrosis [8,9]. 

**Figure 1:**
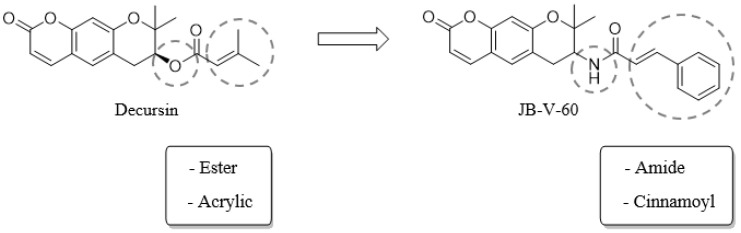
Structure and synthetic strategy of decursin derivative JB-V-60.

Decursin, decursin angelate, and (+)-decursinol, the primary active natural products of the root of *Angelica gigas Nakai*, exhibit various biological activities, including anti-inflammatory, antioxidant, immunomodulatory, and anticancer properties. Additionally, these substances exhibit anti-inflammation and antioxidant effects through HO-1 activation [10]. Decursin inhibits the secretion of pro-inflammatory factors such as iNOS, nitric oxide (NO), prostaglandin E2 (PGE2), IL-6, and TNF-α by modulating the NF-κB pathway [11]. Decursin has a chemical structure that features a dihydropyranocoumarin moiety and an ester functional group. However, the ester group renders decursin chemically and metabolically unstable, leading to low bioavailability and limited pharmaceutical applicability. Therefore, we focused on synthesizing a decursin derivative to overcome these limitations. In this study, we examined the influence of JB-V-60 (Figure 1) on HO-1 and inflammatory markers, including TNF-α, interleukin (IL)-1β, and NO, in human pulmonary artery endothelial cells (HPAECs) in vitro, along with the histological changes in LPS-injected mice in vivo. This study aims to determine the roles of JB-V-60 in activating HO-1 signaling and suppressing inflammatory cytokines and evaluate its potential as a therapeutic agent for inflammatory diseases. 

## 2. Results

### 2.1. Effect of JB-V-60 on iNOS and COX-2 Expression in LPS-Stimulated HPAECs

**Figure 2:**
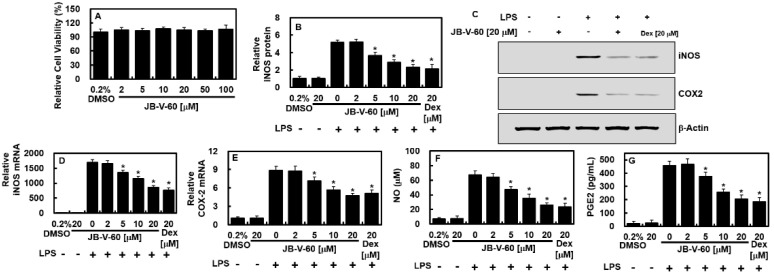
JB-V-60 treatment in LPS-stimulated HPAECs reduces COX-2 and iNOS levels. The effect of JB-V-60 on cell viability was evaluated using the MTT assay (**A**). HPAECs were treated with LPS, followed by exposure to varying concentrations of JB-V-60 or Dex. iNOS protein (**B**,**C**), COX-2 protein (**C**), iNOS mRNA (**D**), COX-2 mRNA (**E**), NO (**F**), and PGE2 (**G**) levels were measured as the outcomes. Data are expressed as the mean ± SD from three independent experiments. * *p* < 0.05 vs. LPS. Abbreviations: HPAECs, human pulmonary artery endothelial cells; LPS, lipopolysaccharide; COX-2, cyclooxygenase; iNOS, inducible nitric oxide synthase; NO, nitric oxide; PGE2, prostaglandin E2; SD, standard deviation.

The MTT assay revealed that JB-V-60 was not cytotoxic to HPAECs, as cell viability remained unaffected at concentrations up to 100 μM (Figure 2A). To investigate the effect of JB-V-60 on the expression of inflammation-associated genes, we examined the levels of iNOS and COX-2, which are key pro-inflammatory markers. HPAECs were initially stimulated with LPS for 6 h and then treated with varying concentrations of JB-V-60 or 20 μM dexamethasone (Dex) for an additional 6 h. Results from a quantitative real-time polymerase chain reaction (qT-PCR), enzyme-linked immunosorbent assays (ELISAs), and immunoblot assays revealed that JB-V-60 and 20 μM Dex significantly reduced LPS-induced iNOS and COX-2 expression in a dose-dependent manner (Figure 2B–E). Additionally, treatment with JB-V-60 or Dex decreased levels of associated molecules PGE2 and NO (Figure 2F,G). These findings indicate that JB-V-60 effectively inhibits iNOS and reduces NO production following LPS stimulation.

### 2.2. Effect of JB-V-60 on NF-κB Activity, STAT-1 Phosphorylation, and HO-1 Protein Levels in LPS-Stimulated HPAECs

**Figure 3:**
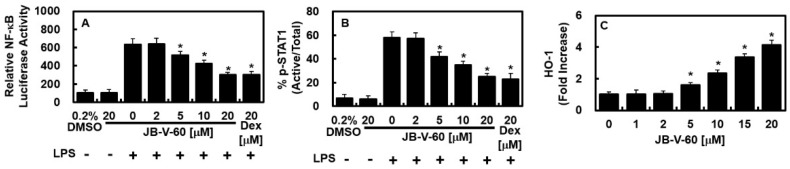
JB-V-60 inhibits the activities of NF-κB and STAT-1 while promoting an increase in HO-1 protein levels. In LPS-stimulated HPAECs, treatments with both JB-V-60 and Dex reduce NF-κB and STAT-1 activities and elevate HO-1 protein levels. (**A**) NF-κB activity was measured using the NF-κB luciferase reporter assay, (**B**) STAT-1 phosphorylation was assessed via ELISA, and (**C**) HO-1 levels were also quantified through ELISA. Results are presented as mean ± SD from three separate experiments. * *p* < 0.05 vs. LPS. Abbreviations: HPAECs, human pulmonary artery endothelial cells; LPS, lipopolysaccharide; STAT-1, signal transducer and activator of transcription 1; HO-1, heme oxygenase; Dex, dexamethasone; NF, nuclear factor; ELISA, enzyme-linked immunosorbent assay; SD, standard deviation.

Subsequently, we examined the influence of JB-V-60 on the regulation of NF-κB, a key factor in driving the expression of inflammation-associated genes. The results showed that JB-V-60 inhibited NF-κB luciferase reporter activity in a dose-dependent manner (Figure 3A). Given the well-established role of the Janus kinase (JAK)/STAT signaling pathway in regulating LPS-induced iNOS and COX-2 production [12,13], we also evaluated the effect of JB-V-60 on STAT-1 phosphorylation and its downstream targets. These findings indicate that JB-V-60 effectively reduces STAT-1 phosphorylation and its downstream signaling (Figure 3B) while significantly upregulating HO-1 expression (Figure 3C).

### 2.3. Effects of JB-V-60 on Nrf2 Nuclear Translocation, ARE Reporter Activity, and Anti-Inflammatory Mechanisms in LPS-Stimulated HPAECs

**Figure 4:**
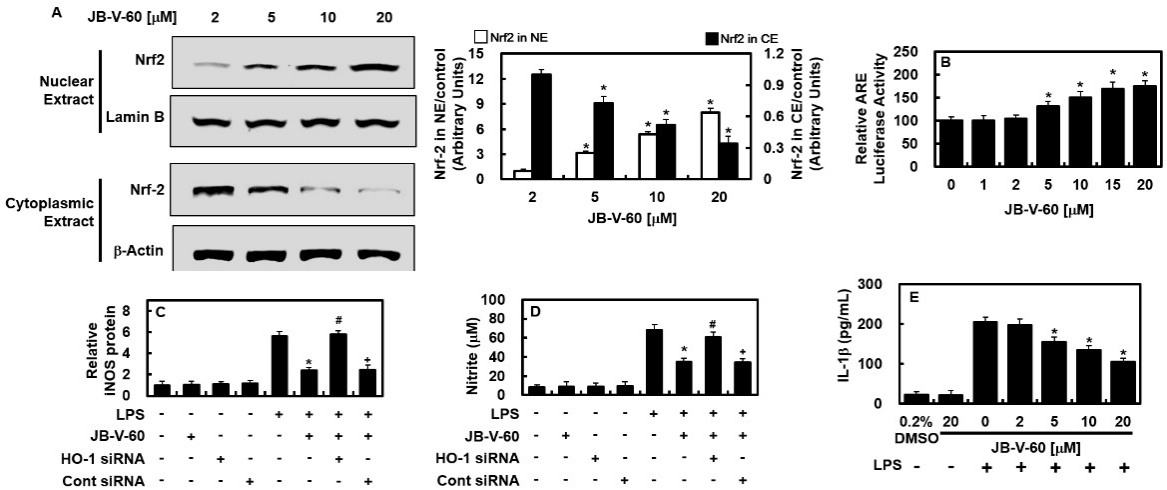
In HPAECs, JB-V-60 promotes the nuclear translocation of Nrf2 and exhibits anti-inflammatory properties. (**A**) Exposure of HPAECs to various JB-V-60 concentrations, followed by the separation and analysis of cytosolic and nuclear fractions for Nrf2 levels via Western blot. (**B**) The activity of the ARE luciferase reporter was assessed in lysates from cells transfected with the ARE construct. (**C**–**E**) HO-1 was knocked down using siRNA, and IL-1β concentrations were measured using an ELISA kit. Results are presented as mean ± SD from three separate experiments. * *p* < 0.05 vs. LPS, # *p* < 0.05 vs. LPS + JB-V-60, or + *p* < 0.05 vs. LPS + JB-V-60 + HO-1 siRNA. Abbreviations: HPAECs, human pulmonary artery endothelial cells; LPS, lipopolysaccharide; siRNA, small interfering RNA; HO-1, heme oxygenase; IL, interleukin; ARE, antioxidant response element; ELISA, enzyme-linked immunosorbent assay; NE, nuclear extract; CE, cytoplasmic extract; SD, standard deviation.

We assessed the role of JB-V-60 in modulating Nrf2 nuclear translocation and ARE-driven gene expression, as Nrf2 is crucial for regulating HO-1 and other antioxidant proteins. The results showed that JB-V-60 facilitated Nrf2 nuclear translocation and significantly enhanced ARE luciferase reporter activity (Figure 4A,B). To elucidate the role of HO-1 in the inhibition of JB-V-60 on iNOS expression, we used small interfering RNA (siRNA) to knock down HO-1 expression. This knockdown restored iNOS and NO levels compared to those of untreated cells, indicating a link between HO-1 upregulation and iNOS reduction (Figure 4C,D). Additionally, JB-V-60 demonstrated anti-inflammatory properties by reducing IL-1β expression in LPS-stimulated HPAECs (Figure 4E).

### 2.4. Effects of JB-V-60 on TNF-α and iNOS Protein Expression in a Mouse Model of LPS-Induced Lung Injury

**Figure 5:**
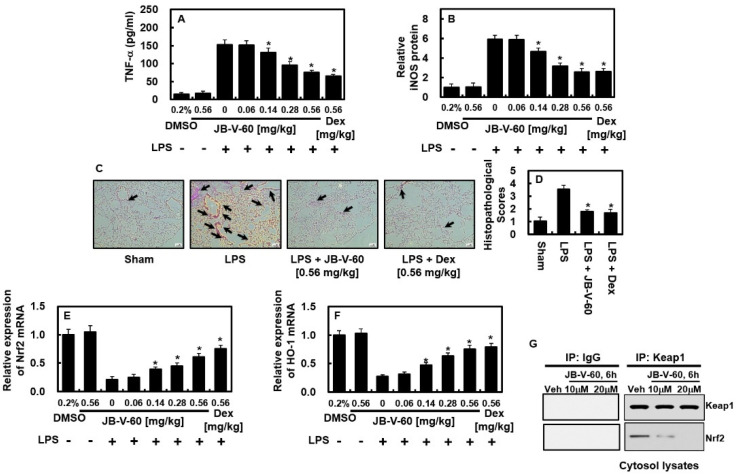
In mice subjected to LPS injection, JB-V-60 effectively reduces TNF-α and iNOS levels while mitigating lung tissue damage. This involves an initial intraperitoneal LPS injection, followed by intravenous administration of JB-V-60 or Dex after 6 h. Lung tissues and BALF were collected 24 h post-LPS injection to assess protein levels of TNF-α (**A**) and iNOS (**B**), as well as mRNA levels of Nrf-2 (**E**) and HO-1 (**F**). The lung tissue was examined via H&E staining (**C**), with histopathological scoring performed (**D**). Arrows indicate leukocyte infiltration. Additionally, the interaction between Keap1 and Nrf2 was analyzed using Co-IP (**G**). Results are expressed as mean ± SD from three independent experiments. * *p* < 0.05 vs. LPS. Abbreviations: LPS, lipopolysaccharide; HO-1, heme oxygenase; Dex, dexamethasone; BALF, bronchoalveolar lavage fluid; iNOS, inducible nitric oxide synthase; Co-IP, co-immunoprecipitation; SD, standard deviation.

The anti-inflammatory properties of JB-V-60 were further examined using an animal model. Figure 5A illustrates that LPS-induced TNF-α levels in the bronchoalveolar lavage fluid (BALF) were significantly decreased in mice treated with JB-V-60 or Dex at a dose of 0.56 mg/kg. Considering the estimated blood volume of mice (72 mL/kg) [14,15] and their average body weight (27 g), the administered doses of JB-V-60 at 0.06, 0.14, 0.28, and 0.56 mg/kg corresponded to final peripheral concentrations of approximately 2, 5, 10, and 20 μM, respectively. In lung tissues, iNOS expression significantly reduced following treatment with JB-V-60 or Dex, indicating the in vivo anti-inflammatory potential of JB-V-60 (Figure 5B). Histopathological analysis further confirmed that JB-V-60 and Dex effectively mitigated LPS-induced lung damage (Figure 5C,D). To further explore the effects of JB-V-60 on the Nrf2/HO-1 pathway and its downstream mediators, we analyzed the expression levels of Nrf2 and HO-1 mRNA in lung tissues. Additionally, we assessed the activities of CAT and SOD, which are downstream mediators of the Nrf2/HO-1 pathway. In LPS-treated mice, JB-V-60 significantly upregulated LPS-suppressed Nrf2 and HO-1 mRNA expression and restored the activities of antioxidant enzymes (Figure 5E,F, Table 1). Furthermore, we investigated whether JB-V-60 affects the Nrf2/Keap1 pathway, thereby inducing HO-1 expression and reducing inflammation. The data showed that treatment with JB-V-60 (10 μM and 20 μM for 6 h) disrupted the Keap1-Nrf2 interaction in primary cells (Figure 5G).

## 3. Discussion

This study demonstrated that LPS stimulation increases inflammatory mediators (NO, PGE2, TNF-α, and IL-1β) and regulatory enzymes (iNOS and COX-2). However, JB-V-60 effectively mitigates this increase, highlighting its anti-inflammatory and antioxidant capabilities in LPS-treated cells and mice. During inflammation, activated endothelial cells release inflammatory mediators (NO, PGE2, TNF-α, and IL-1β) and regulatory enzymes (iNOS and COX-2) [16,17]. However, JB-V-60 significantly reduced these inflammatory markers and enzymes in LPS-stimulated cells and mice, reinforcing its anti-inflammatory and antioxidant potential. Additionally, JB-V-60 increased HO-1 expression in a dose-dependent manner while it reduced LPS-induced COX2/PGE2 and iNOS/NO levels, as well as inhibiting NF-κB activity. NF-κB regulates key inflammatory processes, including cell adhesion, proliferation, differentiation, and the inhibition of apoptosis [18]. Additionally, NF-κB plays a central role in immune responses by activating pro-inflammatory signaling during inflammation. Elevated NO levels are associated with airway inflammation, influencing chemokine production, while NF-κB activation is crucial for LPS-induced COX-2 and iNOS expression. Our findings suggest that JB-V-60 reduces HO-1 production and suppresses the expression of pro-inflammatory mediators (iNOS, COX2, IL1-β, and NO) by modulating NF-κB activity. 

The anti-inflammatory potential of JB-V-60 has been extensively explored in animal models, yielding promising results. We focused on assessing the effects of JB-V-60 on TNF-α and iNOS protein levels in a mouse model of LPS-induced lung injury. The findings indicate a significant reduction in TNF-α levels in the BALF of JB-V-60-treated mice, highlighting its potent anti-inflammatory properties. Additionally, JB-V-60 significantly lowered iNOS expression levels in lung tissues, confirming its efficacy in decreasing inflammation in vivo. Furthermore, JB-V-60 showed protective effects on lung tissues by reducing LPS-induced pulmonary damage. These findings highlight the potential of JB-V-60 as a treatment for inflammatory diseases, especially those affecting the lungs. The study also provides valuable data on the peripheral fluid concentrations of JB-V-60, offering crucial insights for future research and aiding in determining appropriate doses for potential therapeutic use in humans. Overall, this study demonstrates the in vivo anti-inflammatory effects of JB-V-60, particularly in lung inflammation, suggesting its potential for developing new treatments for various inflammatory conditions. However, further research is needed to fully elucidate the mechanisms underlying the anti-inflammatory effects of JB-V-60 and evaluate its suitability for clinical use. 

This study revealed that JB-V-60 enhanced the nuclear translocation of Nrf2 (Figure 4), a crucial step in activating the Nrf2/Keap1 pathway. Evidence of this activation includes the enhanced binding of Nrf2 to AREs after treatment with JB-V-60. Although this does not directly confirm an interaction between JB-V-60 and Nrf2 or Keap1, it strongly suggests that JB-V-60 influences the Nrf2/Keap1 pathway, potentially by stabilizing Nrf2 or inhibiting Keap1. Furthermore, this study showed that JB-V-60 induces the expression of HO-1, a well-known downstream target of the Nrf2 pathway (Figure 3), further supporting the modulation of the Nrf2/Keap1 axis by JB-V-60. The anti-inflammatory effects mediated through HO-1 reinforce the role of this pathway. Moreover, our findings confirmed that JB-V-60 disrupts the Keap1-Nrf2 interaction in HPAECs when treated with 10 μM and 20 μM JB-V-60 for 6 h (Figure 5G). 

Future studies should focus on identifying the specific molecular pathways through which JB-V-60 enhances HO-1 expression and modulates the Nrf2 and NF-κB signaling cascades. Long-term research is also necessary to assess the safety profile and potential adverse effects of JB-V-60, particularly with prolonged use. Refining the dosage and delivery methods of JB-V-60 for various inflammatory conditions is also crucial. Moreover, investigating the effectiveness of JB-V-60 in treating other inflammation-related disorders beyond lung injury, such as arthritis and neuroinflammation, would be valuable. A comparative analysis with current anti-inflammatory treatments could provide insight into how JB-V-60 integrates into the existing therapeutic options. Finally, advancing to clinical trials in humans is crucial for evaluating the pharmacokinetics, pharmacodynamics, and overall clinical potential of JB-V-60, ensuring its development as a viable therapeutic option. 

In conclusion, JB-V-60 effectively elevates HO-1 levels and reduces the production of pro-inflammatory cytokines in LPS-stimulated HPAECs while also decreasing iNOS and TNF-α levels in the lung tissues of LPS-treated mice. These findings underscore the critical role of HO-1 in regulating inflammatory responses and suggest that TNF-α may play a key role in mediating this pathway. Therefore, JB-V-60 emerges as a promising therapeutic candidate for managing inflammatory conditions, particularly those affecting the respiratory system.

## 4. Materials and Methods

### 4.1. Cell Culture and Substances

Primary HPAECs sourced from Cambrex BioScience (C2517AT25; Charles City, IA, USA) were utilized and cultured as previously described [19]. Dex (serving as a positive control), LPS (from *Escherichia coli*), penicillin G, streptomycin, and dimethyl sulfoxide (DMSO) were purchased from Sigma Chemical Co. (St. Louis, MO, USA). Human HO-1 (sc-35554) and control siRNA (sc-37007) were obtained from Santa Cruz Biotechnology (Santa Cruz, CA, USA). HPAECs—between passages 3 and 5—were seeded at a density of 1 × 10⁵ cells per 35 mm dish, then serum-starved overnight for the ELISA. To evaluate HO-1 levels, some cells were treated with LPS (1 μg/mL for 6 h) followed by treatment with JB-V-60 for an additional 6 h, while others were treated with JB-V-60 for 6 h without prior LPS exposure.

### 4.2. General Information of JB-V-60

We synthesized JB-V-60 through the replacement of the ester group with an amide functional group to enhance chemical and metabolic stability. We also replaced the dimethylacrylic group of decursin with a cinnamoyl substituent, as cinnamic acid and its derivatives exhibit antioxidant effects (Figure 1) [20]. All reagent-grade chemicals used were purchased from Sigma-Aldrich (St. Louis, MO, USA), TCI (Tokyo, Japan), and Alfa Aesar (Haverhill, MA, USA). The synthetic starting compound 1 (decursinol) was purchased from Dasan Pharmaceutical Co., Ltd. (Seoul, Republic of Korea). All reactions were performed under an inert atmosphere of dry nitrogen conditions using dry solvents. The reactions were monitored with TLC analysis on silica gel F-254 thin-layer plates. Compounds on the TLC plates were visualized under UV light (254 nm). Flash column chromatography was performed on silica gel 60 (235–400 mesh). Melting points were measured using an Electrothermal IA 9000 melting point apparatus without correction. ^1^H and ^13^C NMR spectra were recorded on a Bruker AVANCE Neo 400 (400 MHz). Chemical shifts are reported in ppm (δ) units relative to the undeuterated solvent, which serves as the reference peak (CDCl_3_—d1: 7.26 ppm/^1^H NMR, 77.16 ppm/^13^C NMR; DMSO—d6: 2.50 ppm/1H NMR, 39.52 ppm/^13^C NMR). The following abbreviations represent NMR peak multiplicities: s (singlet), d (doublet), t (triplet), m (multiplet), dd (doublet of doublets), dt (doublet of triplets), dq (doublet of quartets), td (triplet of doublets), quin (quintuplet), and br (broad signal). Low-resolution mass spectral data were acquired using an Agilent InfinityLab LC/MSD XT, while high-resolution mass spectral data were obtained with a Sciex 1290 Infinity II/TripleTOF 5600 Plus. The final purity of the compound was analyzed using a Shimadzu LC-20 HPLC system (Figures S1–S8).

#### Method for the Synthesis of N-(8,8-dimethyl-2-oxo-7,8-dihydro-2H,6H-pyrano[3,2-g]chromen-7-yl)cinnamamide (JB-V-60) (Scheme 1)

Decursinone (compound 2): A solution of compound 1 (decursinol) (1.00 g, 4.06 mmol) and Dess–Martin periodinane (2.58 g, 6.09 mmol) in CH_2_Cl_2_ (25 mL) was stirred for 5 h at 0 °C. Following 13 h of stirring, the reaction mixture was quenched with saturated NaHCO_3_ aqueous solution and extracted three times with CH_2_Cl_2_ (3 × 15 mL). The combined organic layer was dried with anhydrous Na_2_SO_4_, filtered, and concentrated in vacuo. The residue was purified through column chromatography on silica gel (CH_2_Cl_2_), yielding compound 2 (0.97 g, 98%) as a white solid. ^1^H NMR (400 MHz, CDCl_3_): *δ* 7.64 (d, *J* = 9.5 Hz, 1H), 7.22 (s, 1H), 6.97 (s, 1H), 6.31 (d, *J* = 9.5 Hz, 1H), 3.66 (s, 2H), 1.45 (s, 6H); ^13^C NMR (101 MHz, CDCl_3_): *δ* 208.19, 160.74, 156.08, 154.66, 142.86, 127.10, 119.33, 114.60, 114.55, 106.79, 83.31, 38.73, 24.11; (s); Mp 163.1 °C; LCMS (ESI): *m*/*z* [M + H]^+^ 245.1.

7-((4-methoxybenzyl)amino)-8,8-dimethyl-7,8-dihydro-2H,6H-pyrano[3,2-g]chromen-2-one (compound 3): A solution of compound 2 (250 mg, 1.02 mmol), 4-methoxylbenzylamine (204 μL, 1.56 mmol), and acetic acid (64 μL, 1.12 mmol) in THF (5 mL) was stirred for 10 min at rt. The crude reaction mixture was then added to NaBH_3_CN (192 mg, 3.06 mmol) and stirred for 18 h at the same temperature. Following 18 h of stirring, the reaction mixture was quenched with H_2_O and extracted three times with EtOAc (3 × 10 mL). The combined organic layer was dried with anhydrous Na_2_SO_4_, filtered, and concentrated in vacuo. The residue was purified via column chromatography on silica gel (n-hexane/EtOAc = 3:1, *v*/*v*), yielding compound 3 (338 mg, 91%) as a white solid. ^1^H NMR (400 MHz, CDCl_3_): *δ* 7.59 (d, *J* = 9.5 Hz, 1H), 7.28 (s, 1H), 7.26 (s, 1H), 7.17 (s, 1H), 6.88 (d, *J* = 8.5 Hz, 2H), 6.76 (s, 1H), 6.22 (d, *J* = 9.4 Hz, 1H), 3.93 (d, *J* = 13.1 Hz, 1H), 3.82 (s, 3H), 3.77 (d, *J* = 13.1 Hz, 1H), 3.04 (dd, *J* = 16.0, 4.6 Hz, 1H), 2.81 (dd, *J* = 7.9, 4.9 Hz, 1H), 2.71 (dd, *J* = 16.0, 8.0 Hz, 1H), 1.42 (s, 3H), 1.34 (s, 3H); ^13^C NMR (101 MHz, CDCl_3_) *δ* 161.40, 158.78, 157.01, 154.17, 143.20, 132.38, 129.20, 128.72, 117.74, 113.84, 113.01, 112.56, 104.59, 79.20, 56.18, 55.29, 51.33, 28.10, 26.51, 21.54; Mp 103 °C; LCMS (ESI): *m*/*z* [M + H]+ 366.1.

7-amino-8,8-dimethyl-7,8-dihydro-2H,6H-pyrano[3,2-g]chromen-2-one (compound 4): A solution of compound 3 (298 mg, 0.82 mmol), DDQ (279 mg, 1.23 mmol) in CH_2_Cl_2_ (6 mL), and H_2_O (375 μL) was stirred for 21 h at rt. After stirring for 21 h, the reaction mixture was quenched with saturated NaHCO_3_ aqueous solution and extracted three times with CH_2_Cl_2_ (3 × 10 mL). The combined organic layer was dried over anhydrous Na_2_SO_4_, filtered, and concentrated in vacuo. The residue was purified using column chromatography on silica gel (CH_2_Cl_2_/MeOH = 20:1, *v*/*v*), yielding compound 4 (141 mg, 70%) as a yellowish semisolid. ^1^H NMR (400 MHz, CDCl_3_): *δ* 7.58 (d, *J* = 9.5 Hz, 1H), 7.16 (s, 1H), 6.76 (s, 1H), 6.21 (d, *J* = 9.5 Hz, 1H), 3.10–3.00 (m, 2H), 2.68–2.58 (m, 1H), 1.40 (s, 3H), 1.30 (s, 3H); ^13^C NMR (101 MHz, CDCl_3_): *δ* 161.42, 156.81, 154.14, 143.25, 128.66, 117.61, 113.08, 112.66, 104.58, 78.97, 51.30, 31.30, 25.99, 20.90; Mp not applicable; LCMS (ESI): *m*/*z* [M + H]+ 245.1.

N-(8,8-dimethyl-2-oxo-7,8-dihydro-2H,6H-pyrano[3,2-g]chromen-7-yl)cinnamamide (5; JB-V-60): A solution of compound 4 (104 mg, 0.42 mmol), trans-cinnamic acid (74 mg, 0.50 mmol), EDC∙HCl (113 mg, 0.59 mmol), TEA (88 μL, 0.63 mmol), and 4-DMAP (12 mg, 0.10 mmol) in CH_2_Cl_2_ (2 mL) was stirred for 3 h at rt. After stirring for 3 h, the reaction mixture was quenched with saturated NH_4_Cl aqueous solution and extracted three times with CH_2_Cl_2_ (3 × 10 mL). The combined organic layer was dried with anhydrous Na_2_SO_4_, filtered, and concentrated in vacuo. The residue was purified via column chromatography on silica gel (n-hexane/EtOAc = 2:1, *v*/*v*), yielding compound 5 (JB-V-60) (127 mg, 80%) as a white solid. ^1^H NMR (400 MHz, CDCl_3_): *δ* 7.64 (d, *J* = 15.6 Hz, 1H), 7.56 (d, *J* = 9.5 Hz, 1H), 7.46 (dd, *J* = 6.6, 2.8 Hz, 2H), 7.36 – 7.29 (m, 3H), 7.18 (s, 1H), 6.75 (s, 1H), 6.43 (d, *J* = 15.6 Hz, 1H), 6.18 (d, *J* = 9.5 Hz, 1H), 6.12 (d, *J* = 9.2 Hz, 1H), 4.52 – 4.44 (m, 1H), 3.24 (dd, *J* = 17.3, 4.8 Hz, 1H), 2.86 (dd, *J* = 17.1, 2.6 Hz, 1H), 1.47 (s, 3H), 1.38 (s, 3H); ^13^C NMR (101 MHz, CDCl_3_): *δ* 165.77, 161.18, 156.50, 154.09, 143.12, 141.76, 134.67, 129.81, 129.38, 128.80, 127.84, 120.21, 116.03, 113.48, 113.23, 105.07, 77.78, 48.01, 28.84, 24.93, 24.89; Mp 216–218 °C; HRMS (ESI-TOF): *m*/*z* calculated for C_23_H_21_NO_4_ [M + H]+: 376.1543, found: 376.1548; The purity of JB-V-60 was confirmed through HPLC analysis (Figure S9), which indicated a purity of 99.8% using a Shim-pack GIS-ODS C-18 column (150 × 4.6 mm, 5 μm, Figures S1–S8). The mobile-phase conditions were established using an isocratic solvent system, with solvents A (acetonitrile) and B [0.1% H_3_PO_4_ (*v*/*v*) in water]. The isocratic elution was performed as follows: from 0 to 15 min, 60% A. The flow rate of the mobile phase was 1.0 mL/min, with detection occurring at 256 nm. The column temperature was 40 °C, and the injection volume was 10 μL.

### 4.3. In Vitro Studies

#### 4.3.1. Cell Viability Assay

To evaluate cell viability, the MTT assay was conducted according to previously described methods [21,22]. HPAECs were seeded at a density of 5 × 10^3^ cells per well in 96-well plates and exposed to JB-V-60 for 48 h. After treatment, the cells were rinsed and incubated for an additional 4 h with 100 µL of MTT solution (1 mg/mL) added to each well. The resulting formazan crystals were dissolved in 150 µL of DMSO, and the absorbance was measured at 540 nm using a spectrophotometer (Tecan, Austria GmbH). The viability of the treated cells was determined as a percentage relative to the control group, which was set at 100%.

#### 4.3.2. Enzyme-Linked Immunosorbent Assays (ELISAs)

To assess the effects of JB-V-60 on HPAECs, the cells were initially treated with LPS (1 μg/mL) for 6 h, followed by a 6 h exposure to JB-V-60. Additionally, a separate group of cells was treated with JB-V-60 for 6 h without prior LPS stimulation to evaluate HO-1 levels. STAT1 phosphorylation was measured using ELISA kits provided by Abcam (ab126455, Cambridge, MA, USA), while the concentrations of PGE2, HO-1, IL-1β, TNF-α, and iNOS were determined using ELISA kits from R&D Systems, analyzing the resulting supernatant from the cell cultures after centrifugation.

#### 4.3.3. Nitrite Levels

NO production was quantified by measuring nitrite (NO2-) levels in the cell culture medium. This was achieved by mixing equal volumes of the supernatant with Griess reagent (ab234044, Abcam) and incubating the mixture at room temperature for 15 min. The absorbance of the resulting solution was measured at 540 nm using a spectrophotometer. The entire process was repeated thrice to ensure the reliability and reliability of the results.

#### 4.3.4. Intracellular Fractionation, Co-Immunoprecipitation (Co-IP), and Immunoblotting

After cell collection, the supernatant was isolated via centrifugation, followed by the preparation of cytosolic and nuclear extracts using previously described methods [23]. Immunoblotting was performed using antibodies specific to iNOS, COX-2, lamin B, Keap1, Nrf2, and β-actin, all sourced from Santa Cruz Biotechnology (CA, USA). Nuclear and cytosolic extracts were normalized using Lamin B and β-actin, respectively, as loading controls. The Keap1-Nrf2 interaction in HPAECs was examined using a co-immunoprecipitation (Co-IP) assay with protein A-agarose. Briefly, HPAECs were lysed, and the lysate was centrifuged at 4 °C to separate the supernatant, which was subsequently treated with protein A-agarose to reduce nonspecific binding. After 5 min of centrifugation, the resulting supernatant was incubated with anti-Nrf2 or IgG for 1 h at 4 °C, followed by the addition of protein A-agarose. The mixture was shaken overnight, after which the immunoprecipitates were centrifuged, and the supernatant was discarded. Subsequently, the pellets were washed thrice with lysis buffer. Finally, the pellets were suspended in an SDS loading buffer, boiled for 10 min, and then subjected to Western blot analysis using the specified antibodies.

#### 4.3.5. Quantitative Real-Time Polymerase Chain Reaction

RNA was extracted using TRI Reagent (Invitrogen) and subsequently converted to cDNA in a 20 µL reaction mix that included 0.5 mg/µL of the oligo (dT)-adapter primer (Invitrogen) and M-MLV reverse transcriptase (Invitrogen), all processed with the PX2 Thermal Cycler (Thermo Scientific). For qRT-PCR, the levels of iNOS, COX-2, Nrf2, and HO-1 were compared to β-actin, with the specific primer sequences used detailed, including the following: 5′-CCC CAT TAG CAG CCA GTT-3′; COX-2 reverse: 5′-CAT TCC CCA CGG TTT TGA-3′; iNOS forward: 5′-GTT CTC AGC CCA ACA ATA CAA GA-3′; iNOS reverse: 5′-GTG GAC GGG TCG ATG TCA C-3′; Nrf2 forward: 5′-TCC TAT GCG TGA ATC CCA AT-3′; Nrf2 reverse: 5′-GCG GCT TGA ATG TTT GTC TT-3′; HO-1 forward: 5′-GGG CTG TGA ACT CTG TCC AAT-3′; HO-1 reverse: 5′- GGT GAG GGA ACT GTG TCA GG-3′; β-actin forward: 5′-TCG TGC GTG ACA TCA AAG A-3′; and β-actin reverse: 5′-CAT ACC CAA GAA GGA AGG CT-3′.

#### 4.3.6. Transfection

Cells were co-transfected with the NF-κB-luciferase reporter, HO-1 siRNA, ARE luciferase reporter plasmids, and control siRNA using SuperFect (Qiagen, CA, USA). After a 4 h incubation period with the plasmids, the cells were supplied with fresh growth medium.

#### 4.3.7. ARE Luciferase Reporter Assay

The cells were carefully rinsed with PBS at ambient temperature and subsequently lysed with the buffer from the dual luciferase kit (Promega, WI, USA). Luciferase activity was then quantified using a TD-20/20 luminometer (Turner Designs, CA, USA). Each transfection was conducted in triplicate, with separate experiments, with results expressed as the ratio of firefly to Renilla luciferase activity.

### 4.4. In Vivo Studies

#### 4.4.1. Mouse Model of LPS-Induced Lung Injury

Male C57BL/6 mice, aged 6–7 weeks old with an average weight of 27 g, were procured from Orient Bio Inc. (Seongnam, Republic of Korea). Before the experiments, the mice underwent a 12-day acclimation period to ensure adequate adjustment, as referenced in previous studies [24,25]. LPS was administered intraperitoneally at a dose of 15 mg/kg, with 0.2% DMSO serving as the vehicle control, using 28-gauge needles. Six h after administering the LPS injection, the mice were administered an intravenous injection of JB-V-60 at doses ranging from 0.06 to 0.56 mg/kg (n = 10 per group). The study protocol was approved by the Animal Care Committee of Kyungpook National University (IRB No. KNU 2022-112). BALF was obtained through gentle suctioning following intratracheal administration of PBS. The fluid was then centrifuged at 3000 rpm for 10 min at 4 °C. The resulting supernatant was collected and stored at −80 °C for future analysis.

#### 4.4.2. Histopathological Analysis

Ten mice were administered an intraperitoneal injection of LPS, followed by intravenous administration of JB-V-60 (0.56 mg/kg) 6 h later. The mice were subsequently euthanized, and lung tissues were histologically examined using H&E staining, following the procedures outlined in previous studies [26]. Pulmonary damage was evaluated and scored on a scale of 1–4 based on a predefined grading system [23].

#### 4.4.3. Evaluation of Oxidative Stress Markers

Superoxide dismutase (SOD) activity was assessed using an SOD assay kit (Fluka, Japan) and reported in units per milligram of protein. Catalase activity (CAT) was measured using a CAT assay kit (Sigma Aldrich) based on the rate of H_2_O_2_ breakdown, and the results were presented in units per milligram of protein.

### 4.5. Statistical Analysis

Data are presented as average values with standard deviation (SD) derived from three separate trials. To compare groups, one-way ANOVA was applied, followed by Tukey’s post hoc analysis to identify significant differences, with statistical significance set at a *p*-value < 0.05.

## Figures and Tables

**Scheme 1:**
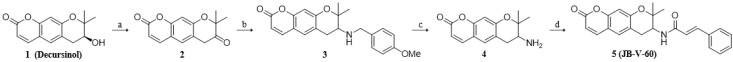
Synthesis of decursin derivative 5 (JB-V-60)**.** Reagents and conditions; (a) Dess–Martin periodinane (1.5 equiv), CH_2_Cl_2_, 0 °C, 5 h; (b) (i) 4-methoxylbenzylamine (1.5 equiv), acetic acid (1.1 equiv), tetrahydrofuran (THF), room temperature (rt), 10 min; (ii) NaBH_3_CN (2 equiv), rt, 18 h; (c) 2,3-dichloro-5,6-dicyano-1,4-benzoquinone (DDQ) (1.5 equiv), CH_2_Cl_2_, H_2_O, rt, 21 h; (d) trans-cinnamic acid (1.2 equiv), 1-ethyl-3-(3-dimethylaminopropyl)carbodiimide hydrochloride (EDC·HCl) (1.4 equiv), triethylamine (TEA) (1.5 equiv), 4-dimethylaminopyridine (4-DMAP) (0.8 equiv), CH_2_Cl_2_, rt, 3 h.

**Table 1:** Effects of JB-V-60 treatment on the activities of antioxidant enzymes in LPS-injected mice.

	SOD (U/mg Protein)	CAT (U/mg Protein)
DMSO	1.05 ± 0.03	4.03 ± 0.15
JB-V-60 (0.56 mg/kg)	1.04 ± 0.02	4.11 ± 0.11
LPS	0.57 ± 0.03	2.63 ± 0.23
LPS + JB-V-60 (0.06 mg/kg)	0.52 ± 0.04	2.71 ± 0.12
LPS + JB-V-60 (0.14 mg/kg)	0.65 ± 0.04 *	3.18 ± 0.15 *
LPS + JB-V-60 (0.28 mg/kg)	0.72 ± 0.03 *	3.73 ± 0.21 *
LPS + JB-V-60 (0.56 mg/kg)	0.91 ± 0.04 *	3.95 ± 0.21 *

Each value represents the mean ± SD (n = 10). * *p* < 0.05 vs. LPS.

## Data Availability

Data is contained within the article and Supplementary Materials.

## Supplementary Materials

The following supporting information can be downloaded at https://www.mdpi.com/article/10.3390/ph17101337/s1: Figure S1: ^1^H NMR spectrum of compound 2; Figure S2: ^13^C NMR spectrum of compound 2; Figure S3: ^1^H NMR spectrum of compound 3; Figure S4: ^13^C NMR spectrum of compound 3; Figure S5: ^1^H NMR spectrum of compound 4; Figure S6: ^13^C NMR spectrum of compound 4; Figure S7: ^1^H NMR spectrum of compound 5 (JB-V-60); Figure S8: ^13^C NMR spectrum of compound 5 (JB-V-60); Figure S9: HPLC chromatogram of compound 5(JB-V-60).
