# Supplementary material for: Inhibitory Effects of Decursin Derivative against Lipopolysaccharide-Induced Inflammation
Source: Pharmaceuticals (Basel). 2024 Oct 7;17(10):1337. doi: 10.3390/ph17101337 (PMC11509908; doi:10.3390/ph17101337)
Supplement: Supplementary file 1 [file pharmaceuticals-17-01337-s001.zip › pharmaceuticals-3213596-supplementary.pdf]

Supporting information

The inhibitory effects of decursin derivative against lipopolysaccharide-induced inflammation

Jinhee Lee[1,*], Jong Beom Heo[2,*], Sanghee Cho[1,*], Chang-Woo Ryu[1], Hae Joon Heo[2], Mi-Young Yun[3], Gaewon Nam[4], Gyu Yong Song[2,] and Jong-Sup Bae[1,**]**

[1] College of Pharmacy, Research Institute of Pharmaceutical Sciences, Kyungpook National University, Daegu 41566, Republic of Korea; aadd8563@gmail.com (J.L); sanghee3472@naver.com (S.C); ryu76kr@daum.net (C.-W.R)

[2] College of Pharmacy, Chungnam National University, 99 Daehak-ro, Yuseong-gu, Daejon 34134, Korea; songmeom@gmail.com (J.B.H); boymatt@naver.com (H.J.H)

[3] Department of Beauty Science, Kwangju Women's University, Gwangju 62396, Republic of Korea; beauty@kwu.ac.kr (M.-Y.Y)

[4] Department of Bio-cosmetic Science, Seowon University 377-3, Musimseoro, Seowon-gu, Cheongju, Chungbuk, Korea 28674; skarod@gmail.com (G.N)

[*] These authors contributed equally.

[**] Correspondence: gysong@cnu.ac.kr (G.Y.S) and baejs@knu.ac.kr (J.-S.B.)

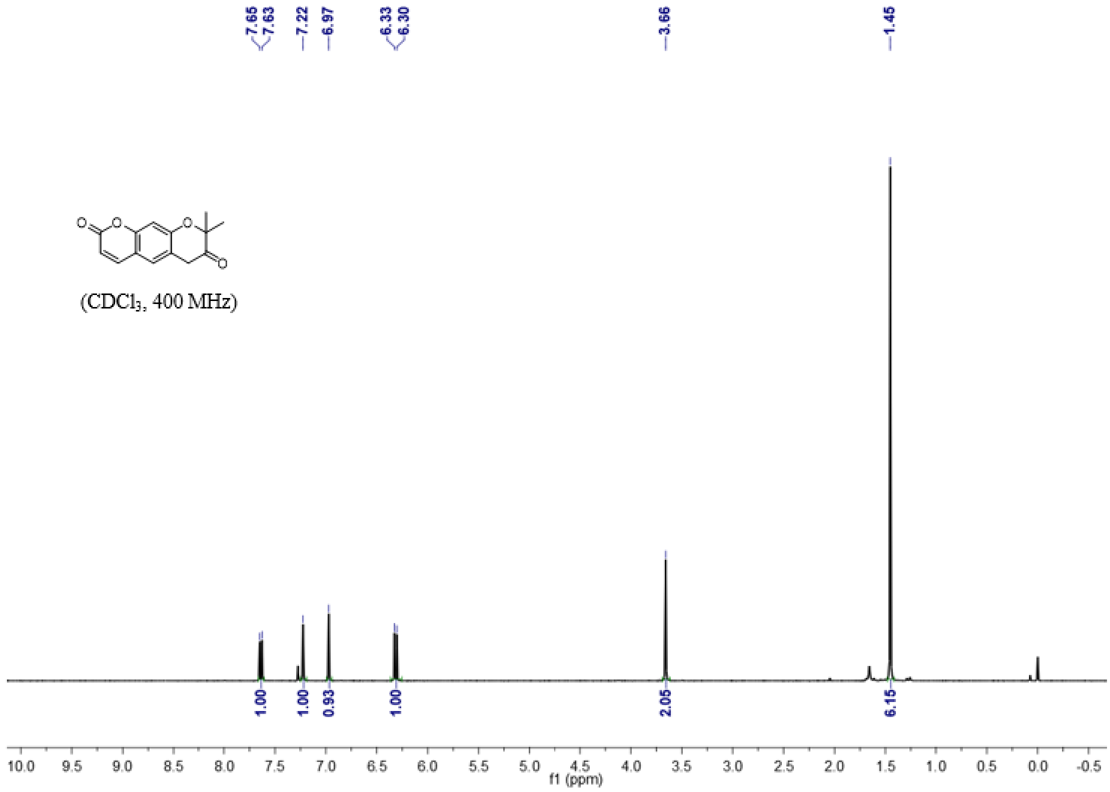

Figure S1. ^{1}H NMR spectrum of compound **2**

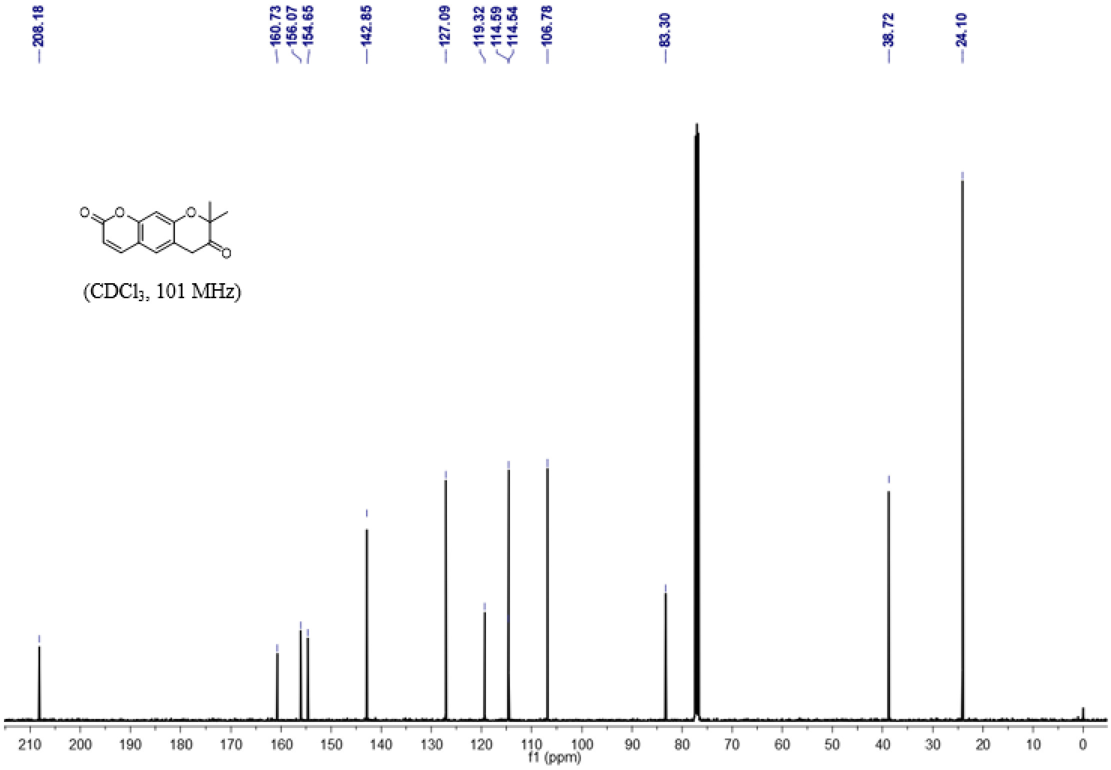

Figure S2. ^{13}C NMR spectrum of compound **2**

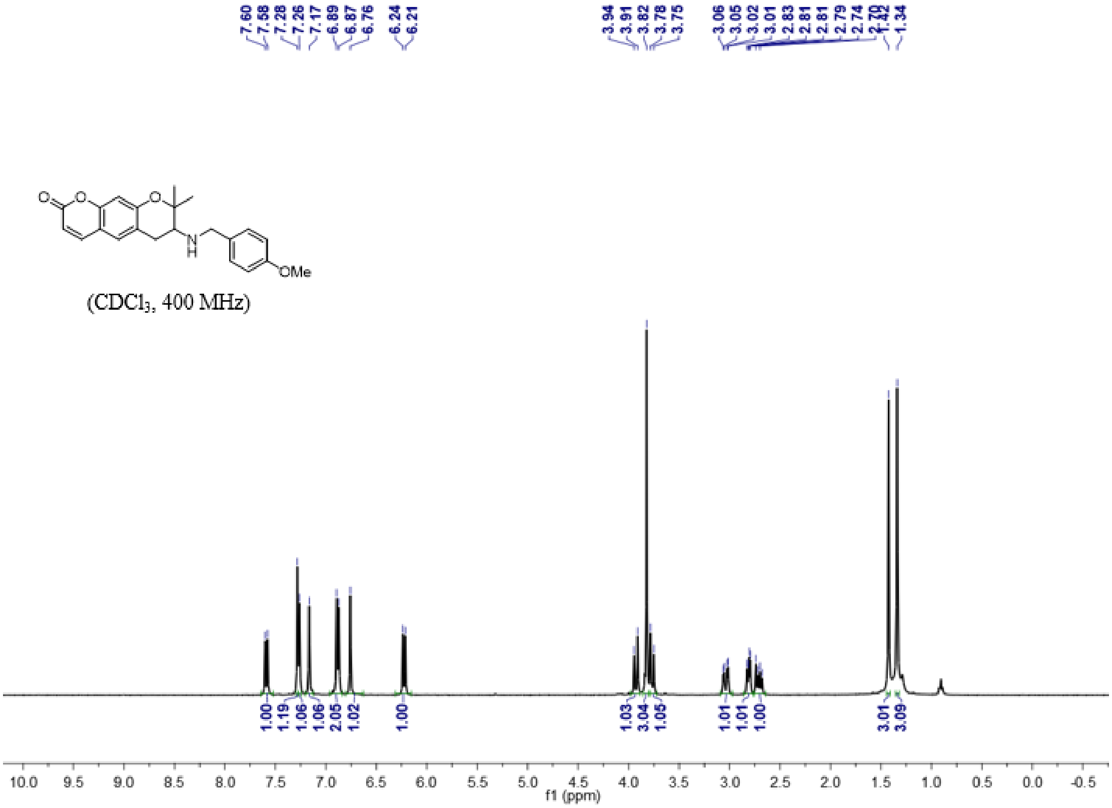

Figure S3. ^{1}H NMR spectrum of compound **3**

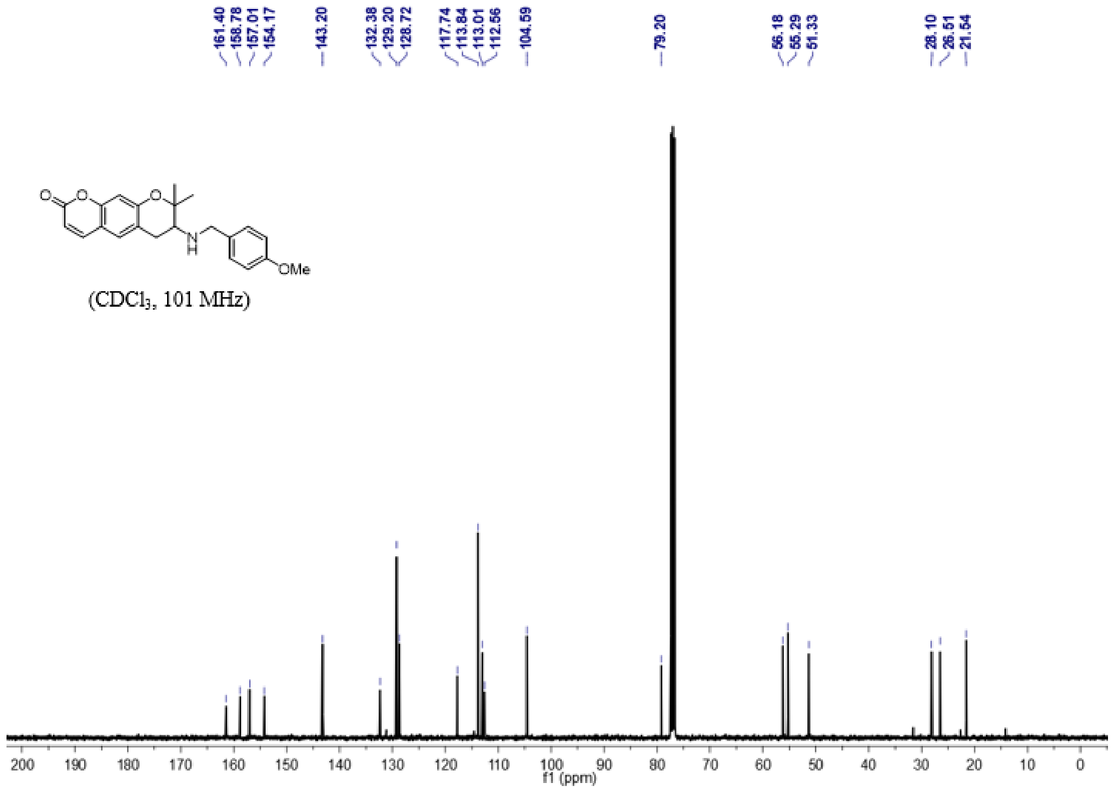

Figure S4. ^{13}C NMR spectrum of compound **3**

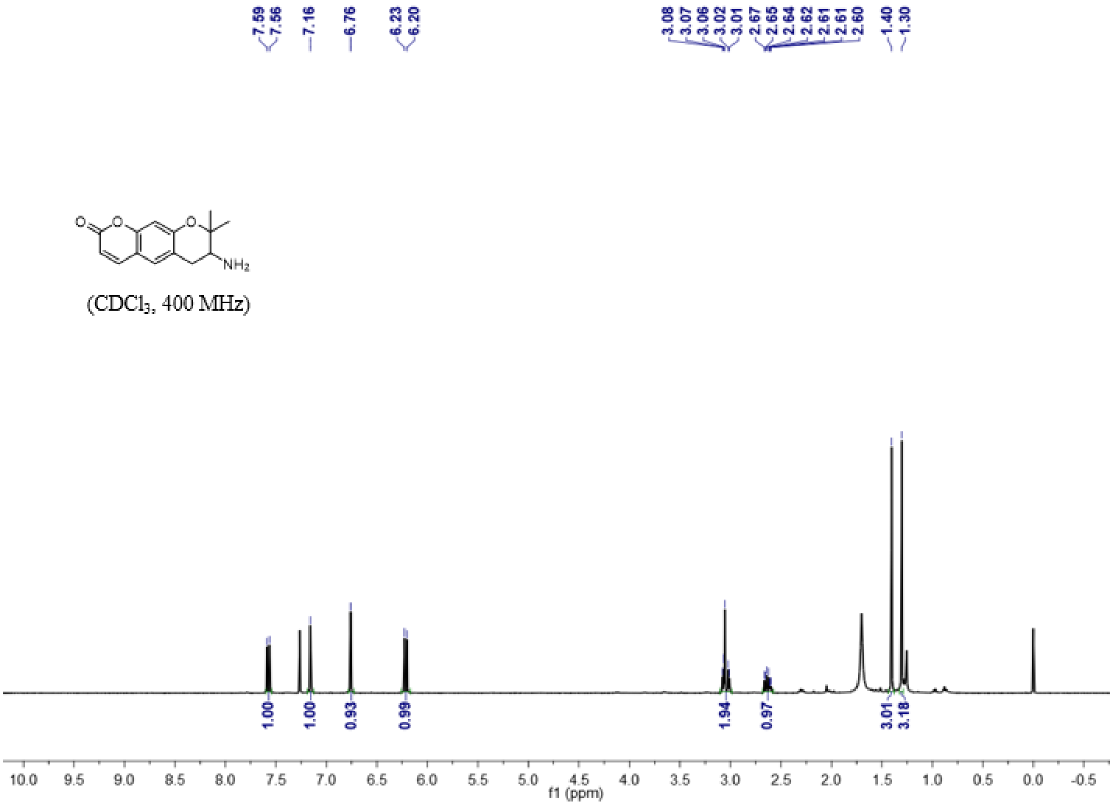

Figure S5. ^{1}H NMR spectrum of compound **4**

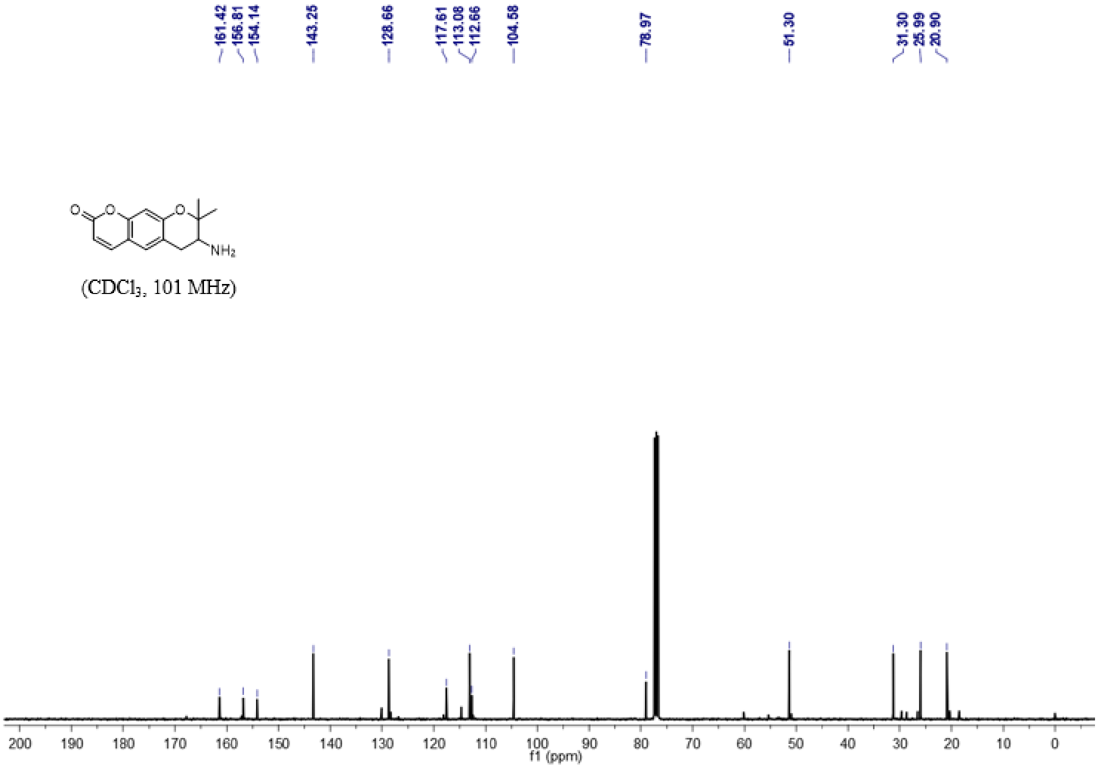

Figure S6. ^{13}C NMR spectrum of compound **4**

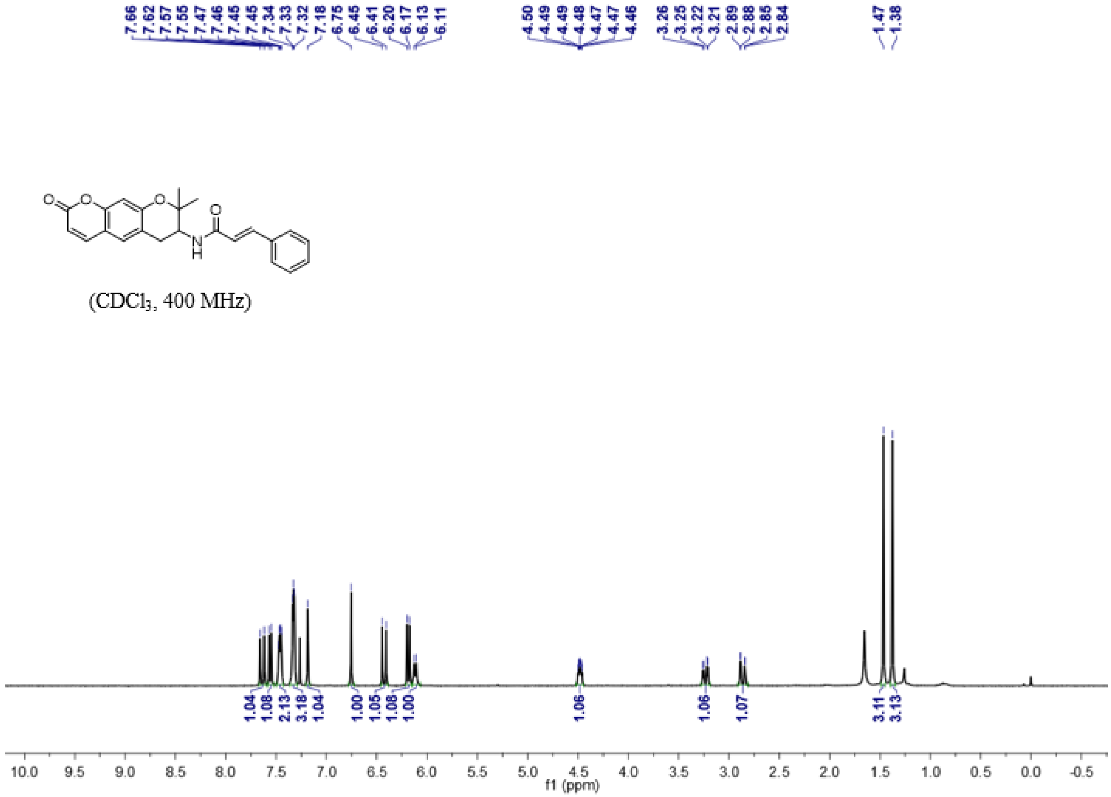

Figure S7. ^{1}H NMR spectrum of compound **5 (JB-V-60)**

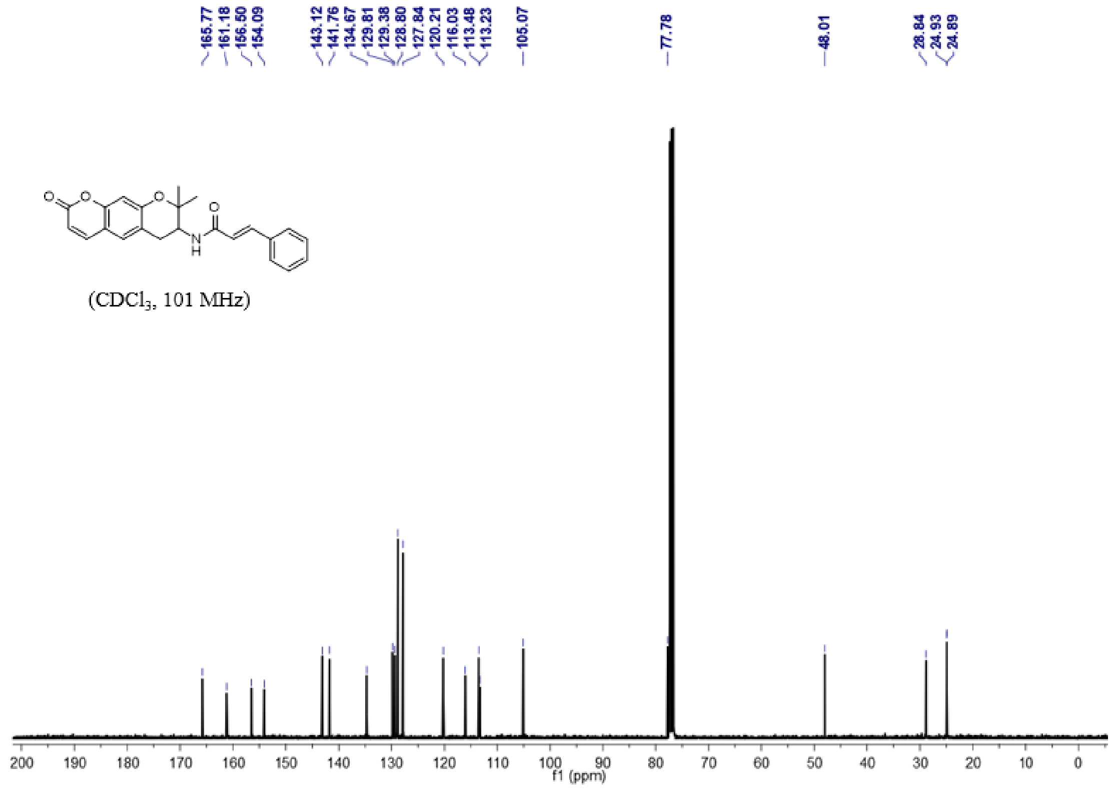

Figure S8. ^{13}C NMR spectrum of compound **5 (JB-V-60)**

<Chromatogram>

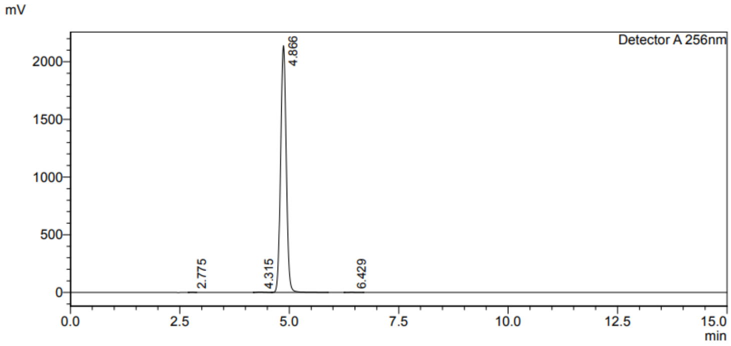

<Peak Table>

Detector A 256nm

Ret. Time	USP Width	Area	Height	Area%
2.775	0.151	4794	937	0.025
4.315	0.309	30766	2714	0.161
4.866	0.242	19108603	2137801	99.785
6.429	0.293	5571	516	0.029
		19149734	2141967	100.000

Figure S9. HPLC chromatogram of compound **5(JB-V-60)**
